# Coupled hydraulic behavior of confluence and diversion flow with multi-gate regulation in a hydropower expansion project

**DOI:** 10.1371/journal.pone.0333502

**Published:** 2025-09-30

**Authors:** Jing Tian, Shiya Guo, Peng Xu, Yu Wang

**Affiliations:** 1 College of Water Conservancy Engineering, Yellow River Conservancy Technical University, Kaifeng, China; 2 Research Center of Disaster Prevention Engineering of Giant Water Network in Henan Province, Kaifeng, China; 3 Advanced Institute of Finance, Henan University, Zhengzhou, China; 4 Henan Engineering Research Center of Project Operation and Ecological Security for Inter-Basin Regional Water Diversion Project, Kaifeng, China; NED University of Engineering and Technology, PAKISTAN

## Abstract

Confluence and diversion flows are common yet complex three-dimensional hydraulic phenomena, and understanding their behavior is crucial for the safe and efficient operation of hydraulic structures, especially when they occur in close proximity within the same project. This study investigated the hydraulic interactions between the culvert intersection and the regulating and diversion gates of a hydropower station expansion using a 1:30 scale physical hydraulic model (Froude similarity). Practical operational strategies for the gates were developed to meet diversion flow requirements under normal conditions. Results indicate that, under typical operational and maintenance scenarios, the Froude number (Fr) between the culvert intersection and the regulating gate ranges from 0.03 to 0.26, indicating that the flow remains subcritical throughout this section. The water level at the Phase I culvert outlet is primarily governed by the total discharge from the Phase I and Phase II culverts, with flow distribution between them has a secondary effect. The stable water level upstream of the regulating gate is minimally influenced by the culvert flow distribution. Moreover, the converging flow has a limited impact on diverging flow at the regulating and diversion gates. The diversion flow rate of the diversion gate remains largely unaffected by the regulating gate’s operation, confirming the proposed gate operation schemes meet the diversion requirements without causing adverse hydraulic interactions.

## 1 Introduction

With the deepening development of hydropower under the ‘carbon peak and carbon neutrality’ goals, hydropower station expansion and renovation projects have become increasingly prevalent [1]. In such projects, the coordinated operation of newly added and existing water conveyance systems often encounters complex hydraulic coupling challenges.

**Fig 1:**
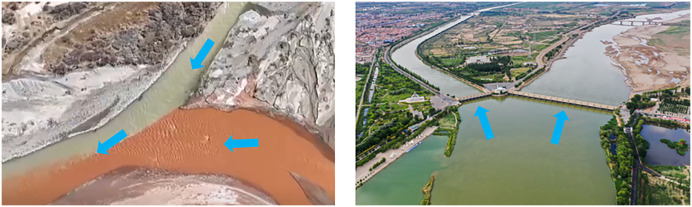
(a) Confluence of a Y-shaped river in Qinghai Province, China; (b) The SanShengGong Hydro-junction on the Yellow River, China.

Confluent and divergent flows are common in both hydraulic engineering and natural river systems (Fig 1). These regions exhibit complex three-dimensional flow structures, particularly near confluence and divergence zones, where turbulence, recirculation, and flow instabilities often occur. Such flow behaviors can significantly impact the operational efficiency and safety of hydraulic structures.

Extensive research has been conducted on confluence and divergence flows. However, most studies have analyzed confluence and divergence separately. Studies on confluence flows have focused on flow structures [2–11], hydrodynamic characteristics [12–24], the effects of confluence angles and flow ratios [25–30], and confluence flow dynamics in non-pressurized tunnels [31–36]. Research on divergent flows has primarily examined flow distribution ratios [37–39], divergence point locations [40–41], hydraulic characteristics of diversion outlets [42], and the effects of boundary conditions, such as divergence angles, on flow characteristics [43,44].

Few studies have explored the coupled hydraulic behavior where confluence and divergence occur in close proximity within the same hydraulic project, leaving an important research gap for hydropower expansion and renovation. Shi et al. [45] studied the variation in hydrodynamic characteristics such as the discharge ratio of the Zeya River and the distributary ratio of the bifurcated river with changing flow rates. Li et al. [46] investigated the influence of river island location and shape at river confluence on the flow distribution ratio of the left sluice, right sluice and weir. However, these studies primarily focus on natural rivers.

Despite extensive research on confluence and divergence flows, a critical gap remains in understanding their coupled hydraulic behavior when both occur in close spatial proximity—a situation increasingly common in modern hydropower renovation and expansion projects. Existing studies typically treat confluence and divergence independently, often focusing on natural river systems rather than water conservancy projects. Through a 1:30-scale physical hydraulic model representing an actual hydropower station renovation project, this study focuses on the hydraulic interaction between Phase I and Phase II culvert flows as they converge into a tailrace channel and then diverge through regulating and diversion gates.The comparative analysis was conducted on the water levels, flow velocities, and flow patterns under various conditions, including different total flow rates and flow distributions in Phase I and Phase II culverts, as well as different discharge rates at the regulating and diversion gates. Based on these results, operation schemes for the diversion and regulating gates were developed to meet diversion flow requirements under typical operating conditions. This research provides valuable insights for the design and management of hydropower station expansion and renovation projects.

## 2 Project overview

**Fig 2:**
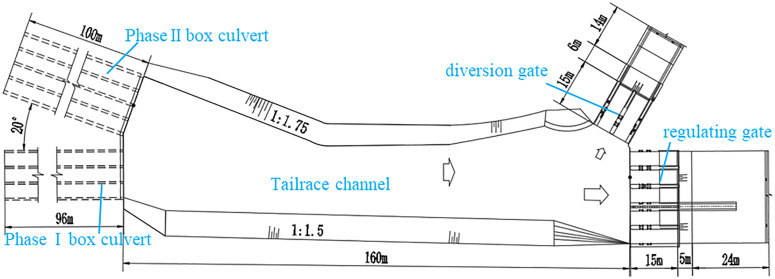
Plan layout of the project.

Phase II of the hydropower station will be constructed using the tailrace of the Phase I station, primarily for the power generation. The project is classified as medium-scale. This study focuses on the hydraulic challenges arising from the integration of Phase II into the existing Phase I system. The tailwater converges after passing through the existing Phase I box culvert (design flow rate: 75 m^3^/s) and the newly constructed Phase II box culvert (design flow rate: 146 m^3^/s) before flowing into the elevated Phase I tailrace channel. This configuration gives rise to complex confluence flow conditions. Following the completion of Phase II, a key concern is whether transitions between free-surface and pressurized flow may occur in the Phase I box culvert, potentially affecting operational safety—issues that are central to the current research. The project layout is shown in Fig 2.

Both the Phase I and Phase II box culverts have a rectangular cross-section with a centerline intersection angle of 20°. The Phase I box culvert is 96 m long, consisting of three cells, each 4 m wide and 4 m high, originally designed for non-pressurized flow. To the left of the Phase I culvert, the newly constructed Phase II box culvert is 100 m long, with four cells, each 4 m wide and 5.5 m high, also designed for non-pressurized flow.

Approximately 160 m downstream of the culvert intersection, the diversion and regulating gates are installed at a centerline intersection angle of 60°. The diversion gate consists of two flat steel gates, each 4.5 m wide, designed for a diversion flow rate of 75 m^3^/s. The original regulating gate has three flat steel gates, each 4.5 m wide. To accommodate maintenance needs for the diversion gate, the discharge capacity of the regulating gate will be increased from 75 m^3^/s to 221 m^3^/s by adding two newly bays on its right side. Two new bays will be equipped with arched steel gates, each 4.5 m wide. The sill elevations of both gates, as well as the channel bed elevation upstream, are set at 1337.8 m. The designed water level at the diversion and regulating gates is 1342.275 m, with a verification water level of 1343.1 m.

Given the variability in flow conditions resulting from different operating scenarios of the power-generating units, determining appropriate operational strategies for the regulating and diversion gates under these new flow conditions is not only an urgent practical issue, but also a central objective of this study.

Operational constraints of the power-generating units in Phases I and II cause variations in total flow and flow distribution within the box culverts, which in turn affect the discharge rates at the regulating and diversion gates. Several key hydraulic questions arise under different operating conditions:

- Do the confluent and divergent flows influence each other under different operating conditions?
- Since the Phase I box culvert was originally designed for non-pressurized flow, will phenomena such as water surface fluctuations and transitions between non-pressurized and pressurized flow occur under different total flow rates and flow distributions?
- How should the diversion and regulating gates be operated to meet the diversion flow requirements?

To address these questions, a hydraulic model test was conducted to study the hydraulic behavior of confluent and divergent flows under various conditions.

## 3 Methodology

**Fig 3:**
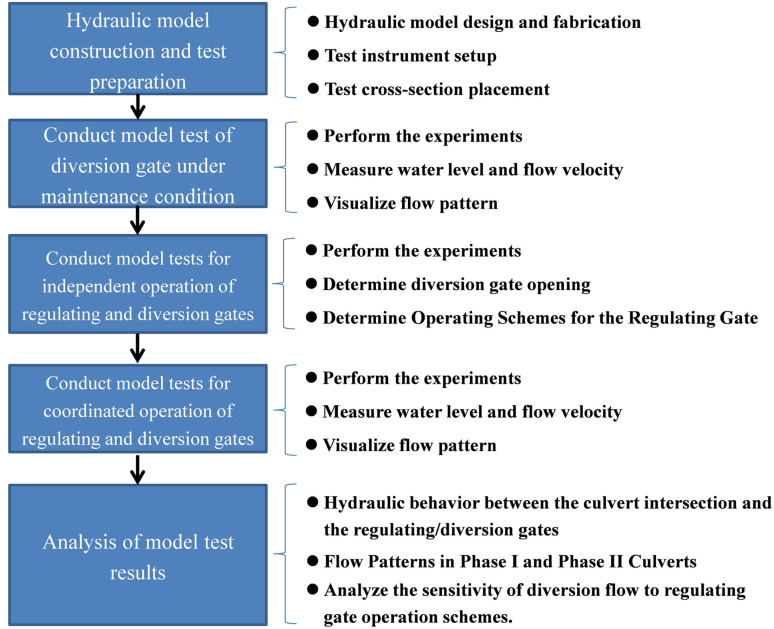
Methodology flowchart.

The research methodology is illustrated in the flow chart below (Fig 3).

### 3.1 Hydraulic model and measurements

The hydraulic model test was conducted at the Water Conservancy Hall of the Yellow River Conservancy Technical University, China. The model was designed based on the gravity similarity criterion with a length scale of 1:30. The key parameters and their scaling factors are listed in Table 1.

**Table 1:** Scaling parameters.

Scale Name	Calculation Formula	Adopted Value
**Geometric scale**	λ_L_	30
**Flow rate scale**	$\lambda_{L}^{\text{5}/\text{2}}$	4929.50
**Velocity scale**	$\lambda_{L}^{\text{1}/\text{2}}$	5.48
**Roughness scale**	$\lambda_{L}^{\text{1}/\text{6}}$	1.76

**Fig 4:**
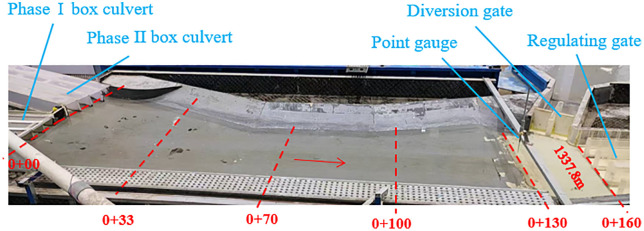
Partial model layout.

The model simulation area included the Phase I and Phase II box culverts, the trapezoidal tailrace channel (main channel), the regulating gate and its downstream channel, and the diversion gate and its downstream channel. To simulate the upstream approach flow, water tanks were installed at the inlets of the Phase I and Phase II box culverts, representing the upstream channels. The Phase I and Phase II box culverts, as well as the diversion and regulating gates, were constructed using transparent plexiglass, while the channels were coated with cement-sand mortar. A 211-meter section downstream of the regulating gate and a 115-meter section downstream of the diversion gate were included in the model to ensure accurate simulation of flow conditions and energy dissipation. The hydraulic model layout is shown in Fig 4.

Six cross-sections (marked by blue lines in Fig 4) were positioned between the culvert intersection and the regulating gates. Cross-sections were denser upstream and downstream of the diversion gate, spaced every 3 meters from the diversion gate to the upstream water level indicator (Fig 4) and every 1.5 meters within the downstream jump pool.

The flow rates of the Phase I and Phase II box culverts were measured using an electromagnetic flowmeter with an accuracy of±0.5%. Water levels were recorded with a movable point gauge with an accuracy of ±0.1 mm, and flow velocities were measured with a propeller current meter with an accuracy of ±2%. The discharge from the diversion gate was determined using a rectangular thin-plate weir located downstream with an accuracy of ±0.1 mm.

### 3.2 Testing scheme and scenarios

The flow rates of the Phase I and Phase II culverts were determined based on the operating conditions of the generator units in Phases I and II of the hydropower station. Considering the functions of the diversion gate (controlled discharge of 75 m³/s) and the regulating gate (which operates when the tailrace channel flow exceeds 75 m³/s), a total of 16 test scenarios were defined for normal operation and maintenance of the diversion gate, as shown in Table 2.

**Table 2:** Test conditions.

Conditions		Flow Rate/(m^3^•s^-1^)		Flow Rate/(m^3^•s^-1^)	
		Phase I	Phase II	Regulating Gate	Diversion Gate
**Normal operation of the diversion gate**	1	37.50	35.50	0.00	73.00
	2	37.50	52.38	14.88	75.00
	3	37.50	108.50	71.00	75.00
	4	37.50	125.38	87.88	75.00
	5	16.88	73.00	14.88	75.00
	6	73.00	73.00	71.00	75.00
	7	16.88	146.00	87.88	75.00
	8	75.00	146.00	146.00	75.00
**Maintenance of the diversion gate**	9	37.50	35.50	73	0.00
	10	37.50	52.38	89.88	0.00
	11	37.50	108.50	146.00	0.00
	12	37.50	125.38	162.88	0.00
	13	16.88	73.00	89.88	0.00
	14	73.00	73.00	146.00	0.00
	15	16.88	146.00	162.88	0.00
	16	75.00	146.00	221.00	0.00

In normal operating condition 1, when the tailrace channel flow is less than 75 m³/s, the regulating gate remains closed, and all flow is discharged through the diversion gate in an uncontrolled manner. In other normal operating conditions, when the tailrace channel flow exceeds 75 m³/s, the diversion gate discharges 75 m³/s, and the excess flow is released through the regulating gate.

Under maintenance conditions, the diversion gate is under repair and does not discharge water. In this case, the entire flow in the tailrace channel is discharged through the regulating gate in an uncontrolled manner.

## 4 Results and analysis

### 4.1 The hydraulic behavior between the culvert intersection and the regulating and diversion gates

When the confluence ratio (Phase II culvert flow rate/ Phase I culvert flow rate) is greater than or close to 1, the Phase II culvert flow dominates after confluence. The main channel flow is biased toward the left bank or remains centrally aligned (when the confluence ratio is close to 1). Flow near the right bank culvert is sluggish.

Under normal operating conditions, the cross-sectional average flow velocity ranges from 0.13 to 1.79 m/s between the culvert intersection and the regulating gate, with the Froude number (Fr) ranging from 0.04 to 0.26. During maintenance conditions, the cross-sectional average flow velocity varies from 0.11 to 1.65 m/s, with Fr ranging from 0.03 to 0.22. In both cases, the flow between the culvert intersection and the regulating gate remains subcritical.

**Fig 5:**
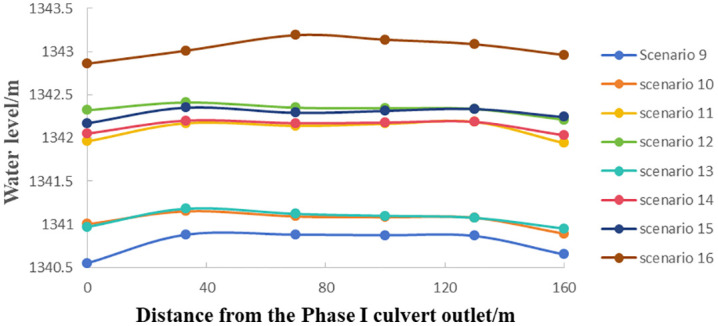
Water surface profile from the Phase I culvert outlet to the regulating gate (maintenance operations).

The water surface profiles reflect flow behavior in the channel. Fig 5 illustrates the water surface profile along the channel from the Phase I culvert outlet to upstream of the regulating gate during maintenance operations. As shown in Fig 5, although the total flow and flow distribution between the Phase I and Phase II box culverts vary under different maintenance conditions, the water surface profile follows a consistent pattern: initially rising, then stabilizing, and finally decreasing. This is because, after confluence, water levels rise due to hydraulic interaction between the two flows. As the combined flow progresses downstream, it gradually becomes uniform, stabilizing the water level. Upon reaching the regulating gate, flow acceleration caused by the narrowing cross-section results in a drop in water level.

The total flow rate of the Phase I and Phase II culverts has the most significant influence on the water surface profile along the channel from the Phase I culvert outlet to the upstream of the regulating gate. Higher total flow rates lead to increased water levels. When the total flow rate remains constant, the flow distribution between the two culverts has little effect on the steady-state water level upstream of the regulating gate.

For example, in scenarios 12 and 15, the total flow rate from both culverts was 162.88 m^3^/s, with the Phase I culvert carrying 23% and 10.4% of the total flow, respectively. However, the water level difference at the measuring point 30 meters upstream of the regulating gate was only 0.06 m. This is because the confluent flow remains subcritical (Fr < 1), allowing the velocity distribution to become uniform along the channel. Consequently, the flow distribution within the culverts has a negligible effect on the stable water level upstream of the regulating gate and a limited impact on divergent flow at the regulating and diversion gates.

### 4.2 Flow patterns in the Phase I and Phase II culverts

The Phase I box culvert features a relatively mild bottom slope (1/32,000). When the outlet water level exceeds the culvert’s top elevation of 1341.61 m, pressurized flow occurs. As shown in Fig 5, the outlet water level of the Phase I culvert is primarily influenced by the total flow rate from both culverts. As the total flow rate increases, the outlet water level rises. The effect of flow distribution is secondary—when the total flow remains constant, a higher proportion of flow in the Phase I culvert results in a higher outlet water level.

For example, in scenarios 12 and 15, both culverts had a total flow rate of 162.88 m^3^/s. However, in scenario 12, the Phase I culvert carried 23% of the flow, whereas in scenario 15, it carried only 10.4%. Consequently, the outlet water level in scenario 12 was 0.15 m higher than in scenario 15.

In scenarios 10 and 11, the Phase I culvert maintained a flow rate of 37.5 m^3^/s, while the Phase II culvert’s flow rate increased from 52.38 m^3^/s (scenario 10) to 108.5 m^3^/s (scenario 11). This increase caused the flow in the Phase I culvert to transition from non-pressurized to pressurized flow. The hydraulic model test showed that as the Phase II culvert flow rate increased, the Phase I culvert transitioned to pressurized flow gradually from downstream to upstream, with minimal surface fluctuations.

This occurs because, after confluence, water levels rise due to hydraulic interaction between the two flows, reducing velocity in the Phase I culvert while increasing its water level. Since the downstream flow remains subcritical (Fr < 1), the flow regime remains stable. Additionally, due to the relatively short culvert lengths, the water level at the Phase I outlet and confluence is highly sensitive to changes in the Phase II culvert flow rate.

Under maintenance condition 16, when both culverts operate at maximum discharge capacity and all flow is released through the regulating gate, the stable water level upstream of the regulating gate reaches 1342.957 m, slightly below the verification water level of 1343.1 m. Air-entraining vortices were observed at the inlet of the Phase II culvert, along with intermittent transitions between non-pressurized and pressurized flow. During actual operation, continuous monitoring is essential to ensure the safe operation of the culverts.

### 4.3 Independent operation of the regulating and diversion gates

Under scenarios 2–8 (Table 2), when the flow rate in the tailrace channel exceeds 75 m^3^/s, the diversion gate discharges 75 m^3^/s, and the remaining flow is released through the regulating gate. To achieve flow diversion, separate operation schemes were established for the diversion and regulating gates, initially disregarding their hydraulic interactions.

#### 4.3.1 Diversion gate operation.

**Fig 6:**
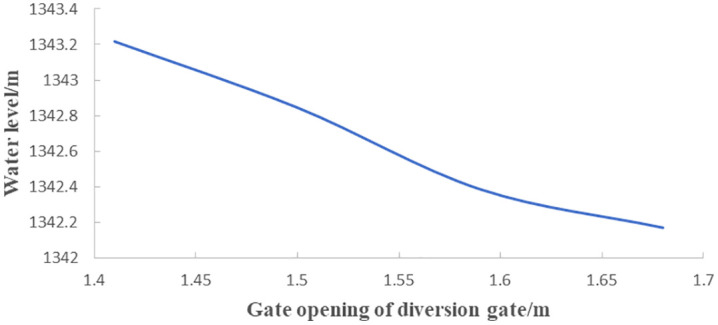
Correlation between the upstream water level and gate opening for diversion gate.

Hydraulic model tests were conducted with the regulating gate closed and a diversion flow of 75 m^3^/s. Fig 6 illustrates the correlation between the upstream water level and the uniform opening of the two diversion gates. According to the curve, when the upstream design water level is 1342.275 m, the required gate opening height for the diversion gate is 1.635 m.

#### 4.3.2 Operation schemes for the regulating gate.

Under scenarios 2–8 (Table 2), the regulating gate operates at four discharge rates: 14.88, 71, 87.88, and 146 m^3^/s. Among the five-bay regulating gates, the two newly added arched steel gates on the right side exhibit lower opening forces and better dynamic stability. Therefore, the operating principle for the regulating gate is to fully open flat gates and use the new arched gates to precisely control the water discharge.

To minimize interference between the diversion and regulating gates, flow should be released preferentially from the regulating gate bays farther from the diversion gate. Hydraulic model tests conducted with the diversion gate closed established a correlation between the upstream water level and the discharge rate for a fully open central bay. At an upstream design water level of 1342.275 m, the central bay discharges 39.2 m^3^/s.

**Fig 7:**
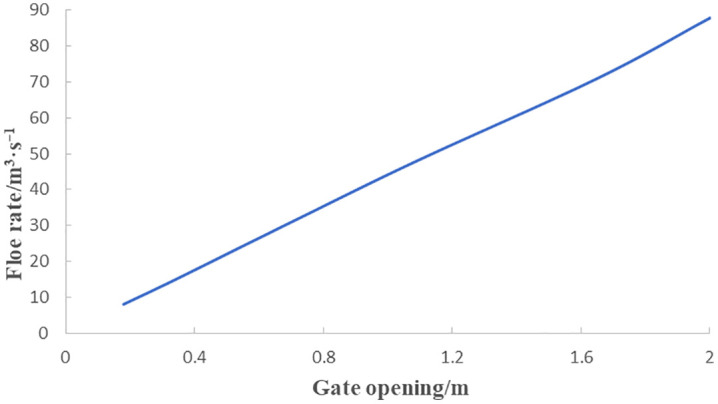
Correlation between flow rate and opening of the two arched gates.

Hydraulic model tests (with the diversion gate closed) show that at an upstream design water level of 1342.275 m, the two new arched steel gates on the right side were opened uniformly. The correlation between the flow rate and the gate opening was demonstrated in Fig 7.

Stable flow patterns are essential for the safe and reliable operation of hydraulic structures. Vortices upstream of gates can cause fluctuating pressure on the gate panels, potentially causing damage to the gate [47]. Backflow can lead to uneven velocity distribution. Through a comparative analysis of flow patterns, operation schemes for the regulating gates under the four discharge rates were formulated as follows:

1. **Discharge Rate of 14.88 m^3^/s**

Since 14.88 m^3^/s is below the 39.2 m^3^/s capacity of a single fully open central bay, the arched steel gates are used for flow release. Two operation schemes are available:

- **Single-gate operation**: Only one arched steel gate is opened.
- **Dual-gate uniform operation**: Both arched steel gates are opened uniformly.

According to Fig 7, the required opening height is 0.34 m for dual-gate operation. If only one arched gate is used, the effective flow width is halved, requiring an estimated gate opening height of 0.68 m.

Flow pattern comparisons reveal that:

- Single-gate operation causes significant velocity variations downstream, inducing pronounced backflow towards the closed gate side.
- Dual-gate operation results in a submerged outflow pattern, preventing backflow.

Thus, the dual-gate uniform opening scheme is recommended for 14.88 m^3^/s discharge rate.

2. **Discharge Rates of 71 m**^**3**^**/s and 87.88 m**^**3**^**/s**

Two operation schemes are considered:

- Dual arched steel gates open uniformly.
- One central bay fully open, with the two arched steel gates opened uniformly.

##### Analysis of dual-gate operation:

**Fig 8:**
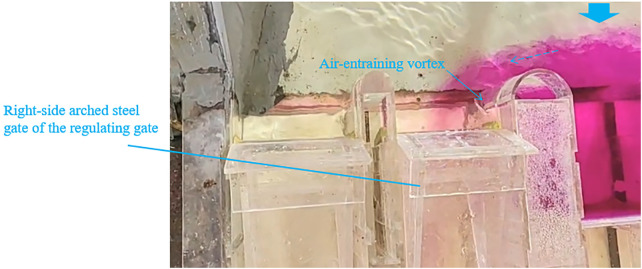
Flow pattern at 71 m^3^/s with two arched gates open.

For a discharge rate of 71 m^3^/s, Fig 7 indicates a required opening height of 1.65 m. For 87.88 m^3^/s, the opening height is 2.0 m. When only the arched steel gates are open while the three flat gates remain closed, high flow velocity occurs upstream of the right-side arched steel gate, generating transverse flow and an air-entraining vortex. At 71 m^3^/s, with a 1.65 m gate opening, the upstream flow pattern is shown in Fig 8. These vortices induce fluctuating pressures on gate panels [47], rendering this scheme unsuitable.

##### Analysis of combined central bay and arched gate operation

For a discharge rate of 71 m^3^/s at 1342.275 m upstream water level, the central bay discharges 39.2 m^3^/s, leaving 31.8 m^3^/s for the arched gates. According to Fig 7, the required arched gate opening is 0.72 m. For 87.88 m^3^/s, 39.2 m^3^/s is discharged through the central bay, with 48.68 m^3^/s through the arched gates, requiring a 1.09 m opening.

**Fig 9:**
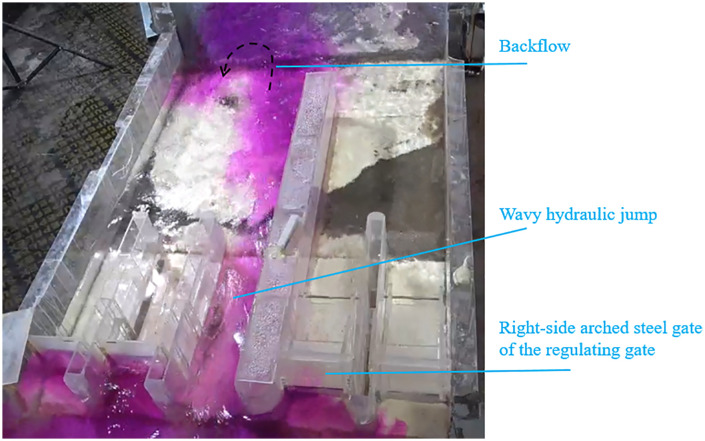
Flow pattern at 87.88 m^3^/s with central bay and two arched gates open.

For both cases, a wavy hydraulic jump and backflow develop in the central bay, but a submerged outflow pattern forms behind the arched gates. At 87.88 m^3^/s, the flow pattern is illustrated in Fig 9. Given its better flow stability, this combined operation scheme is recommended.

3. **Discharge Rate of 146 m^3^/s**

**Fig 10:**
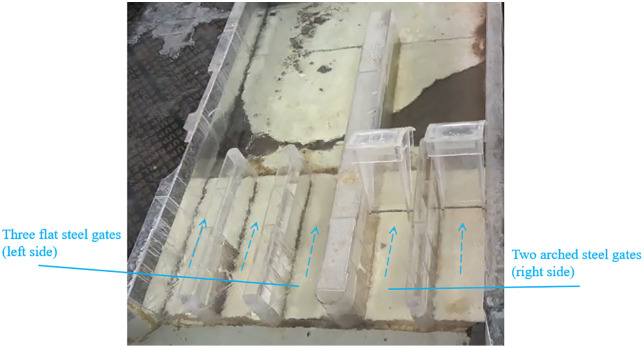
Flow pattern with all five regulating gates open.

At the design discharge of 146 m^3^/s and an upstream water level of 1342.275 m, all five regulating gate bays are fully opened. The resulting flow pattern remains smooth and stable, as shown in Fig 10.

Without considering hydraulic interactions between the regulating and diversion gates, the recommended operation schemes for the regulating gate at discharge rates of 14.88, 71, 87.88, and 146 m^3^/s are summarized in Table 3.

**Table 3:** Operation schemes for the regulating gate.

Discharge Rate(m^3^•s^-1^)	Upstream water level/m	Downstream water level/m	Operation schemes for the regulating gate
14.88	1342.275	1338.4	Both arched steel gates opened to 0.34 m
71	1342.275	1340.4	Central bay fully opened + arched gates at 0.72 m
87.88	1342.275	1340.8	Central bay fully opened + arched gates at 1.09 m
146	1342.275	1341.8	All five bays fully opened

### 4.4 Coordinated operation of regulating and diversion gates

Building on the independent operation and dispatch schemes for the regulating and diversion gates, their mutual hydraulic influence was analyzed, focusing on the sensitivity of the diversion flow rate and upstream flow patterns to different regulating gate operation schemes.

**Fig 11:**
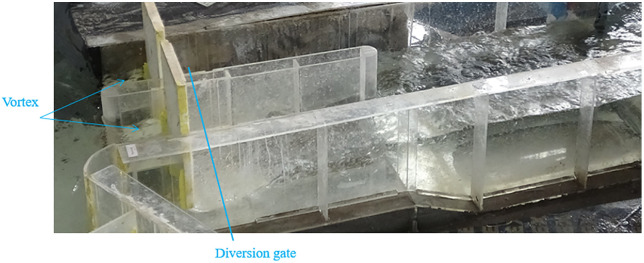
Flow pattern at the diversion gate under scenario 6.

Under normal operating conditions (scenarios 2–8, Table 2), a counterclockwise vortex consistently formed in front of the diversion gate across all regulating gate operation scenarios. This occurs because the flow is deflected leftward, encounters the diversion gate, and is redirected leftward again. The resulting flow pattern at the diversion gate under scenario 6 is shown in Fig 11.

**Fig 12:**
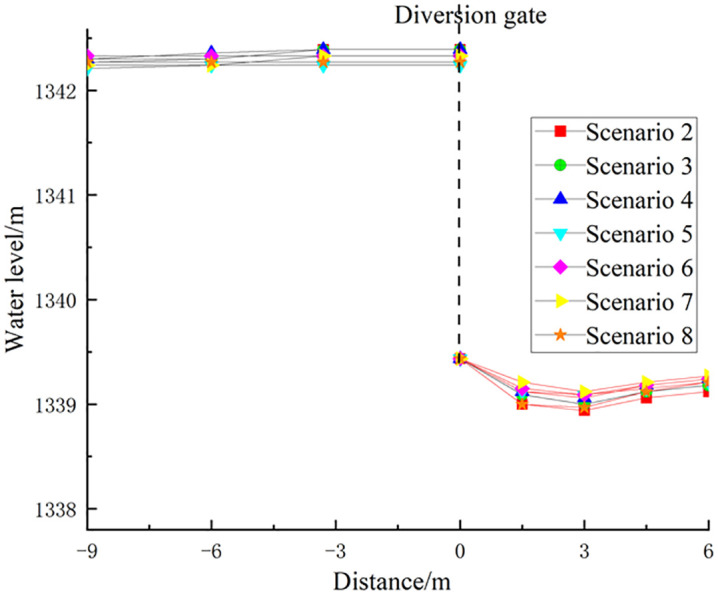
Water level variations upstream and downstream of the diversion gate.

Fig 12 illustrates the water level variations upstream and downstream of the diversion gate for scenarios 2–8 (Table 2). As shown in Fig 12, the upstream water level of the diversion gate remains relatively stable across all normal operating conditions. Flow discharges through the bottom of the diversion gate, forming a contracted section downstream where the flow depth increases before transitioning into the downstream channel via a hydraulic jump.

Measured discharge at the rectangular thin-plate weir under normal conditions ranges from 73.5 to 76.0 m^3^/s, deviating within ±2% of the target 75 m^3^/s. The diversion flow rate is largely insensitive to the regulating gate’s operation, likely due to the strategic selection of regulating gate bays located farther from the diversion gate, effectively minimizing mutual hydraulic interference.

**Fig 13:**
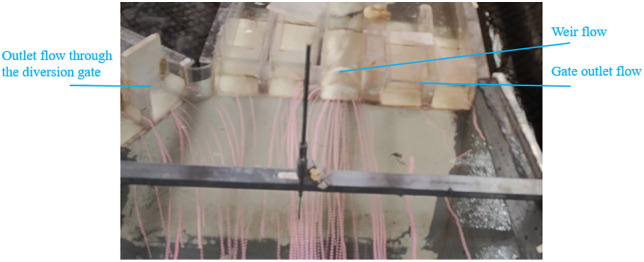
Flow pattern upstream of the regulating and diversion gates under scenario 7.

Fig 13 illustrates the flow patterns upstream of the diversion and regulating gates under scenario 7. As shown in Fig 12, the central bay flow of the regulating gate exhibits weir flow, while the right-side arched gate discharges as a gate outlet flow. On the upstream right side, water rapidly converts potential energy into kinetic energy as it passes through the central bay. At the point gauge 30 m upstream of the regulating gate, the upstream left-side flow is redirected due to the diversion gate’s influence.

Under all operating conditions, water levels at the point gauge 30 m upstream of the regulating gate range from 1342.21 m to 1342.39 m, with minimal variation. This indicates that the regulating gate’s operation has a negligible effect on the diversion gate’s flow rate. Therefore, the operation schemes in Table 3 effectively meet the diversion flow requirements.

## 5 Discussion

In this project, the existing Phase I box culvert originally operates under non-pressurized flow conditions, and the impact of the Phase II box culvert operation on the flow pattern of the Phase I box culvert is one of the key concerns. The flow pattern in the Phase I box culvert is influenced by multiple factors, including culvert length, flow distribution between Phase I and Phase II box culverts, and the total flow rate. In this project, the Phase I box culvert is 96 m long with three cells, while the Phase II box culvert is 100 m long with four cells. Due to the relatively short lengths of both culverts, hydraulic model tests under both normal and maintenance conditions show that the water level response at the Phase I box culvert and the confluence is highly sensitive to changes in the total flow rate of both culverts. This finding is consistent with general observations from culvert hydraulics literature [48], which highlight increased sensitivity in short-channel systems. Consequently, when the total flow rate varies, the transition between non-pressurized flow and pressurized flow in the Phase I culvert is rapid and stable, with minimal flow fluctuations.

The bottom slope of both culverts is 1/32,000, which is also a critical factor influencing the confluence flow and flow patterns in the Phase I box culvert. Due to the mild bottom slope, the transition between non-pressurized flow and pressurized flow in the Phase I culvert is rapid and stable. If the bottom slope were steeper, the flow velocity at the convergence zone and within Phase I would rise accordingly, which could complicate the flow transition and lead to more unstable and complex flow patterns.

The Phase I box culvert is originally designed with a discharge capacity of 75 m^3^/s. Under maintenance conditions, water is discharged through the regulating gate, while under normal operating conditions water is diverted through the diversion gate, resulting in a relatively straightforward initial operation scheme. However, the completion of Phase II significantly increases operational complexity. Under various normal operating conditions with differing flow rates, excess flows beyond the 75 m^3^/s diversion requirement must be discharged through the regulating gate. This gives rise to two primary challenges:

- The discharge capacities of the regulating and diversion gates mutually influence each other, as the discharge capacity of the regulating gate is constrained by the upstream water level, which in turn affects the diversion flow rate.
- The three bays on the left side of the regulating gate are existing flat steel gates, while the two bays on the right side are newly added arched steel gates. Following operational recommendations, the flat gates are generally kept fully open to prolong their service lives, with the newly added arched gates used for precise discharge control. These factors further complicate the coordinated operation of the regulating and diversion gates.

In this study, a stepwise analysis approach was adopted in hydraulic model tests to develop operation schemes for the regulating and diversion gates. Initially, independent operational schemes were developed for the diversion and regulating gates, disregarding their mutual hydraulic influence. Based on flow pattern analysis and the spatial layout of the new and existing regulating gates, the scheme prioritized discharging through bays farther from the diversion gate, which provided a preliminary exploration of optimal independent gate operations. Subsequently, the mutual hydraulic influence between the regulating and diversion gates was incorporated. The model tests demonstrated that across all operating conditions, the developed operation schemes successfully met the diversion flow requirements, while validating the feasibility and effectiveness of the stepwise analysis method in this project.

Although this study successfully developed operation schemes for the regulating and diversion gates through hydraulic model tests, in actual operation, factors such as water quality variations and the aging of gate facilities due to long-term operation can affect flow regulation accuracy. To address these uncertainties, continuous monitoring during actual operation and further research are needed.

This study provides valuable references for the design and operational management of similar hydropower station expansion and renovation projects. The stepwise analysis method for gate operation schemes, based on hydraulic model tests, can be extended to other hydraulic engineering projects under similar conditions.

This study employed hydraulic model tests designed in accordance with the Froude similarity principle. Although scale effects are inherent in hydraulic model tests, the flow in the test region is predominantly turbulent and primarily governed by gravitational forces, which allows for reliable reproduction of overall flow patterns and key hydraulic behaviors. Given the limitations of physical models in spatial and temporal resolution, we intend to incorporate numerical simulation techniques in future work [49]. These simulations will provide more detailed insights into complex flow dynamics, serving as an important complement and extension to the physical model studies.

## 6. Conclusions

Hydraulic model tests were conducted to analyze hydraulic changes following the completion of the Phase II hydropower station, specifically focusing on the confluence flow downstream of the Phase I and Phase II box culverts, the flow division upstream of the diversion and regulating gates, and the flow pattern in the Phase I culvert. The study investigated the complex hydraulic behavior of confluence and diversion between the culvert intersection and the regulating and diversion gates in the Phase I and Phase II projects of a hydropower station. Operation schemes for the diversion and regulating gates were developed to ensure compliance with diversion flow requirements under various normal operating conditions. The results demonstrate that:

The flow from the culvert intersection to the upstream of regulating and diversion gates remains subcritical (Fr < 1). Under different flow distribution conditions between the Phase I and Phase II box culverts, the confluent flow gradually stabilizes along the channel, leading to a more uniform velocity distribution. The impact of flow distribution between Phase I and Phase II culverts on the stable water level upstream of the regulating gate was negligible. Consequently, the confluent flow downstream of the box culvert intersection exerted negligible influence on divergent flow at both the regulating gate and the diversion gate.

After water flow from the Phase I and Phase II culverts converges, the water levels rise due to mutual support between the flows, reducing the flow velocity in Phase I while raising its water level. Under most operating conditions, the Phase I box culvert transitions from non-pressurized flow to pressurized flow. Due to the relatively short lengths of both culverts, the water level response at the Phase I box culvert and the confluence is highly sensitive to changes in the total flow rate. The transition between non-pressurized flow and pressurized flow in the Phase I culvert occurs rapidly and remains stable, with negligible flow fluctuations.

Under all normal operating conditions, the developed operation schemes successfully meet the diversion flow requirements. The scheme prioritizes discharge through the regulating gate bays located farther from the diversion gate, effectively minimizing hydraulic interference between the two gates. In practical operation, the operation schemes for the regulating gate should be further refined based on actual monitoring data.

Future work will focus on numerical simulations of complex flow dynamics near regulating and diversion gates. We aim to enhance practical gate operation strategies by investigating the coordinated control of new and existing regulating gates.

## Supporting information

S1 Data The "Summary of Test Conditions" worksheet provides details for Table 2 (Test Conditions), including the downstream control water levels for the regulating gate and the diversion gates. The "Normal Operation Data" worksheet includes data for normal operation, detailing flow velocities and water surface elevations from the box culvert to downstream of the regulating and diversion gates.The "Maintenance Condition Data" worksheet provides similar data for maintenance conditions. (XLSX)
